# Vascular normalization: reshaping the tumor microenvironment and augmenting antitumor immunity for ovarian cancer

**DOI:** 10.3389/fimmu.2023.1276694

**Published:** 2023-10-23

**Authors:** Ping Yu, Yaru Wang, Dahai Yuan, Yunqin Sun, Shuang Qin, Tianye Li

**Affiliations:** ^1^ Sanquan College of Xinxiang Medical University, Xinxiang, China; ^2^ Department of Radiation Oncology, Hubei Cancer Hospital, Tongji Medical College, Huazhong University of Science and Technology, Wuhan, China; ^3^ Zhejiang Provincial Clinical Research Center for Obstetrics and Gynecology, Hangzhou, China; ^4^ Department of Gynecology, The Second Affiliated Hospital, Zhejiang University School of Medicine, Hangzhou, China

**Keywords:** ovarian cancer, vascular normalization, tumor microenvironment, antitumor immunity, immune infiltration, immunotherapy

## Abstract

Ovarian cancer remains a challenging disease with limited treatment options and poor prognosis. The tumor microenvironment (TME) plays a crucial role in tumor growth, progression, and therapy response. One characteristic feature of the TME is the abnormal tumor vasculature, which is associated with inadequate blood perfusion, hypoxia, and immune evasion. Vascular normalization, a therapeutic strategy aiming to rectify the abnormal tumor vasculature, has emerged as a promising approach to reshape the TME, enhance antitumor immunity, and synergize with immunotherapy in ovarian cancer. This review paper provides a comprehensive overview of vascular normalization and its potential implications in ovarian cancer. In this review, we summarize the intricate interplay between anti-angiogenesis and immune modulation, as well as ICI combined with anti-angiogenesis therapy in ovarian cancer. The compelling evidence discussed in this review contributes to the growing body of knowledge supporting the utilization of combination therapy as a promising treatment paradigm for ovarian cancer, paving the way for further clinical development and optimization of this therapeutic approach.

## 1 Background

According to comprehensive global cancer statistics, ovarian cancer remains a significant health concern for women worldwide. Annually, over 230,000 females receive a distressing diagnosis of ovarian cancer, accounting for approximately 3.6% of newly reported cancer cases. Tragically, the disease claims the lives of more than 150,000 women each year, representing 4.3% of all cancer-related fatalities. Thus, ovarian cancer stands as the second leading cause of death among gynecological malignancies, second only to cervical cancer (1). Ovarian cancer possesses distinctive characteristics, including delayed detection, early metastasis, drug resistance, and challenging treatment protocols, all of which contribute to a poor prognosis (2–4). Within the hierarchy of fatal gynecological tumors, epithelial ovarian cancer claims the highest mortality rate (5–7). Alarming statistics are observed within China, where more than 52,000 women succumb to epithelial ovarian cancer annually, accompanied by a concerning escalation in both incidence and mortality rates (8). However, there is a glimmer of hope in the form of targeted therapies, which have demonstrated promising outcomes as maintenance treatments (9–11). These innovative drugs not only aid in disease control but also offer the potential to delay disease progression and improve the overall prognosis for ovarian cancer patients (12–18).

Among these novel targeted strategies, anti-angiogenesis therapy has become a key issue in the field of cancer therapeutics (19–21). Angiogenesis plays a crucial role in the progression of ovarian cancer, contributing to tumor development and distant metastasis (22). The humanized monoclonal antibody targeting vascular endothelial growth factor (VEGF) bevacizumab, has been extensively investigated in epithelial ovarian cancer (23). The effectiveness of bevacizumab has been confirmed in multiple clinical studies, even in refractory ovarian cancer (24–26). Actually, anti-angiogenesis therapy not only disrupts blood vessels crucial for cancer growth and metastasis but also has a significant impact on reshaping the tumor microenvironment (TME) (27). Preclinical and clinical studies have consistently shown that the combination of anti-angiogenesis with immune checkpoint blockade (ICB) therapy surpasses the efficacy of monotherapy (28–35). In mouse models, the combination therapy demonstrates a remarkable ability to decrease the ratio of pro-tumor to anti-tumor immune cells and effectively downregulate the levels of immune checkpoints, surpassing the effects observed with PD-1 blockade alone (36–39). These findings have generated considerable excitement, leading to the initiation of numerous clinical trials aimed at investigating the synergistic effects of this combination therapy, with encouraging outcomes being observed (40–43). This review provides a comprehensive summary of the most recent understanding surrounding ICB plus anti-angiogenesis strategy, highlighting the significant advances made in relevant clinical trials.

## 2 Anti-angiogenesis therapy for ovarian cancer

### 2.1 Tumor angiogenesis

Angiogenesis, the formation of new blood vessels, was first described by Scottish surgeon John Hunter. It plays a vital role in both normal physiological processes and pathological conditions (44). Physiological angiogenesis occurs during embryogenesis, tissue repair, and regeneration, and is a short-term, controlled process. In contrast, pathological angiogenesis is associated with diseases and involves uncontrolled endothelial cell proliferation, migration, and degradation of the extracellular matrix (45). Then, a groundbreaking study by Folkman revealed the significant relationship between angiogenesis and tumor growth. He introduced the concept that tumor growth depends on neovascularization, marking a milestone in research (46). Tumor angiogenesis primarily occurs through the budding of new blood vessels from existing microvascular beds. These newly formed vessels provide nutrients and oxygen for tumor growth while also serving as a pathway for distant metastasis (47–53). Folkman’s work also led to the emergence of “anti-angiogenesis therapy” as a new approach to cancer treatment and opened up exciting avenues for biomedical research (54–56).

Angiogenesis in tumor neovascularization is regulated by a complex interplay of pro-angiogenic and anti-angiogenesis factors. Pro-angiogenic factors promote angiogenesis, while endogenous inhibitory angiogenic factors prevent excessive vessel formation (57). Examples of pro-angiogenic factors include vascular endothelial growth factor (VEGF), angiopoietin (ANGPT), platelet-derived growth factor (PDGF), fibroblast growth factor (FGF), and other cytokines (58–61). On the other hand, endostatin, angiostatin, platelet factor 4 (PF4), interferon alpha (IFN-α), and other factors act as inhibitory angiogenic factors (62–65). The balance between these factors determines the local angiogenesis of a tumor, with an increase in pro-angiogenic factors or a decrease in anti-angiogenesis factors tipping the balance towards angiogenesis (66).

**Figure 1:**
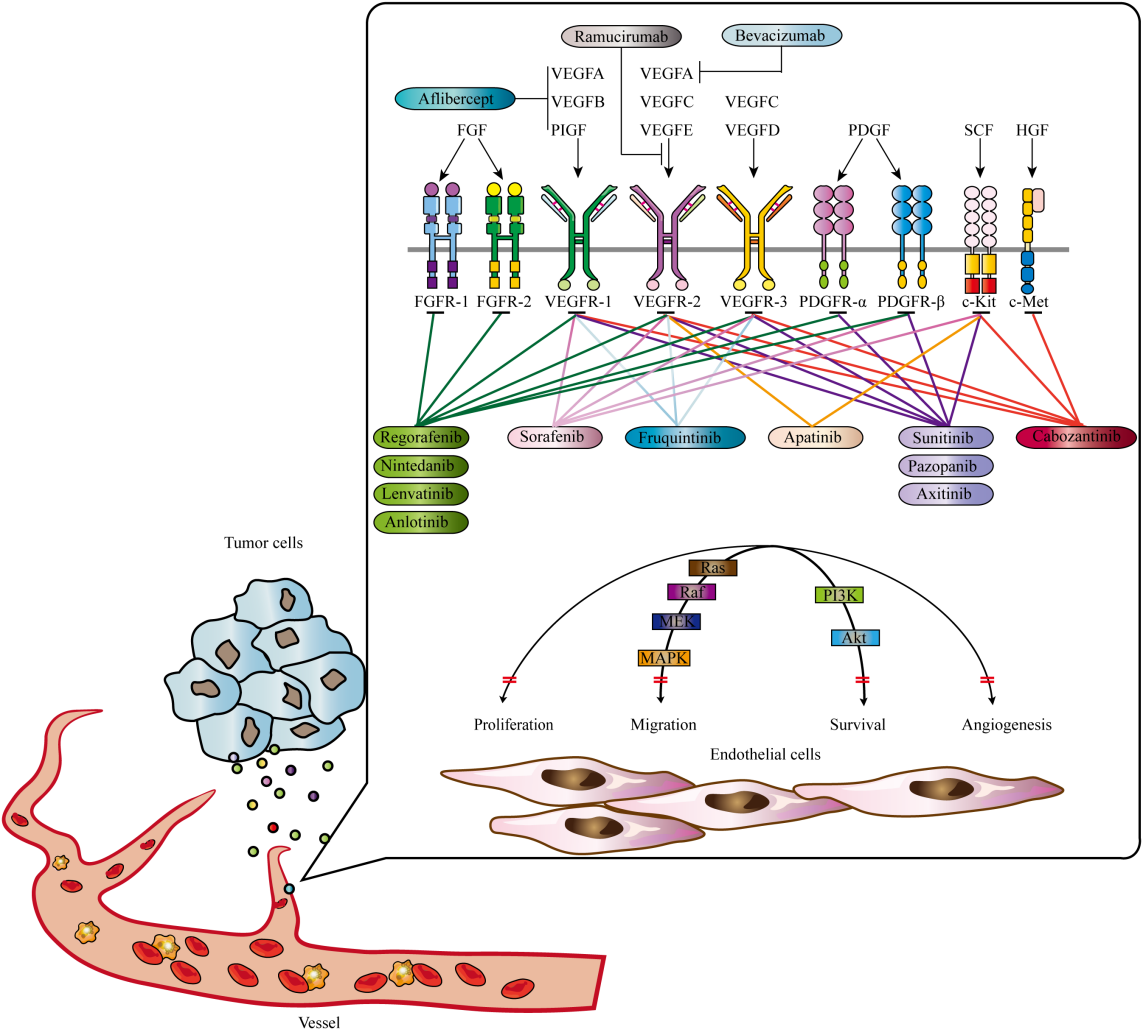
Signaling pathways contributing to tumor angiogenesis and approved anti-tumor angiogenesis drugs. Mutiple signas have been validated to promote tumor angiogenesis, including EGF, FGF, PDGF, HGF, and SCF signaling. Abbrevations: VEGF, Vascular Endothelial Growth Factor; PDGF, Platelet-Derived Growth Factor; FGF, Fibroblast Growth Factor; HGF, Hepatocyte Growth Factor; SCF, Stem Cell Factor. Adapted from Qin et al., 2019 (22).

The VEGF family, consisting of VEGFA, VEGFB, VEGFC, VEGFD, VEGFE, and placental growth factor (PIGF), is a central player in angiogenesis (67). VEGF receptors (VEGFRs), including VEGFR1 (Flt-1), VEGFR2 (KDR/Flk-1), and VEGFR3 (Flt-4), are tyrosine kinase receptors involved in signal transduction (68). Different isoforms of VEGFA exhibit varying levels of activity and localization in the body. VEGFA primarily activates and binds to VEGFR-2, triggering a cascade of intracellular signaling pathways that stimulate endothelial cell proliferation, increase vascular permeability, and promote neo-angiogenesis (69). Another important component in angiogenesis, independent of VEGF/VEGFR, is the ANGPT/TIE system (70). The ANGPT family includes ANGPT1, ANGPT2, ANGPT3, and ANGPT4, with ANGPT1 and ANGPT2 being extensively studied (71). ANGPT1 is mainly expressed in perivascular cells such as pericytes and smooth muscle cells. It interacts with Tie2 receptors in a paracrine manner, promoting endothelial cell viability and maintaining vascular integrity. ANGPT2, predominantly expressed in vascular endothelial cells, competitively blocks the effects of ANGPT1, leading to increased vascular permeability and disruption of the resting vascular system (72–74). ANGPT2’s interaction with endothelial progenitor cells may serve as an initiating factor in neovascularization (75–77). Understanding the mechanisms and factors involved in angiogenesis has significant implications for therapeutic interventions and targeting tumor growth. Anti-angiogenesis strategies aim to inhibit or disrupt the formation of new blood vessels, thereby starving tumors of the necessary nutrients and oxygen for their growth (78). These approaches include the use of anti- angiogenesis drugs that target specific factors or receptors involved in angiogenesis. Generally, angiogenesis is a complex process crucial for normal physiological functions and pathological conditions. It is determined by the delicate balance between anti-angiogenesis and pro-angiogenesis regulators. The VEGF/VEGFR and ANGPT/TIE systems play essential roles in angiogenesis, with VEGFA and ANGPT1 being key players. Intriguingly, nuclear PD-L1 is reported to be implicated in angiogenesis, elucidating the intricate biological signaling that orchestrates the interaction between immune responses and vascularization (79). So far, multiple anti-angiogenesis agents have been approved for cancer treatment **Figure 1**; **Table 1** (80).

**Table 1:** Approved tyrosine kinase inhibitors with anti-angiogenesis capability.

Agents	Targets	Company	Indication	Approval status
**Sorafenib**	VEGFR2/3, RAF, PDGFRβ, FLT3, c-Kit	Bayer and Onyx	RCC	2005 (FDA)
			HCC	2007 (FDA)
			DTC	2013 (FDA)
**Sunitinib**	VEGFR1/2/3, PDGFRα/β, KIT, FLT3, CSF-1R, RET	Pfizer	GIST	2007 (FDA)
			RCC	2007 (FDA)
			pNETs	2011 (FDA)
**Pazopanib**	VEGFR1/2/3, PDGFRβ, c-Kit, FGFR1, c-Fms	GlaxoSmith Kline	RCC	2009 (FDA)
			STS	2012 (FDA)
**Axitinib**	VEGFR1/2/3, PDGFRβ	Pfizer	RCC	2012 (FDA)
**Regorafenib**	VEGFR1/2/3, TIE2, PDGFR-β, FGFR, KIT, RET, RAF	Bayer	CRC	2012 (FDA)
			GIST	2013 (FDA)
			HCC	2017(FDA)
**Cabozantinib**	VEGFR2, MET, RET, AXL, FLT3, c-KIT	Exelixis	MTC	2012 (FDA)
			RCC	2016 (FDA)
**Nintedanib**	VEGFR1/2/3, FGFR1/2/3, PDGFRα/β	Boehringer	IPF	2014 (FDA)
			NSCLC	2014 (EMA)
**Lenvatinib**	VEGFR1/2/3, FGFR1/2/3/4, PDGFR, c-kit, and RET	Eisai	DTC	2015 (FDA)
			RCC	2016 (FDA)
			HCC	2018 (FDA)
**Apatinib**	VEGFR2, RET, c-KIT, Src	Hengrui	GC	2014 (CFDA)
**Anlotinib**	VEGFR2/3, FGFR1/2/3/4, PDGFRα/β, c-Kit, RET	Chia-taiTianqing	NSCLC	2018 (CFDA)
**Fruquintinib**	VEGFR1/2/3	Hutchison	CRC	2018 (CFDA)

CRC, colorectal cancer; DTC, differentiated thyroid cancer; GC, gastric cancer; GIST, gastro-intestinal stromal tumor; HCC, hepatocellular carcinoma; IPF, idiopathic pulmonary fibrosis; MTC, medullary thyroid cancer; NSCLC, non-small cell lung cancer; pNETs, pancreatic neuroendocrine tumors; RCC, renal cell carcinoma; STS, soft tissue sarcoma; CFDA, China Food and Drug Administration; EMA, European Medicines Agency; FDA, United States Food and Drug Administration.

### 2.2 Anti-angiogenesis therapy for ovarian cancer

In the microenvironment of ovarian cancer, VEGF signaling is highly activated and strongly correlated with poor differentiation grade and prognosis (81, 82). Therefore, targeting pro-angiogenic signaling pathways, particularly the VEGF pathway, shows promise as an effective strategy for ovarian cancer. Bevacizumab, the first recombinant humanized anti-VEGF-A antibody, is the most widely used anti-angiogenesis agent (83–85). By inhibiting the binding of VEGF-A to VEGFR, bevacizumab impedes the formation of new blood vessels, interferes pre-existing blood vessels, and downregulates intratumoral microvessel density (86, 87). It has received FDA approval for platinum-sensitive refractory ovarian cancer (88). Besides, it has been also approved by European Commission for advanced epithelial ovarian cancer (89). Bevacizumab has demonstrated clinical efficacy in advanced and recurrent ovarian cancer, resulting in delayed tumor development and serving as a maintenance therapy (90, 91). Combining bevacizumab with chemotherapy has been explored for the treatment of ovarian cancer (92–95). However, recent meta-analysis demonstrates a more pronounced improvement in median progression-free survival (PFS) achieved by bevacizumab alone or in combination with other inhibitors, yet it does not indicate any overall survival benefit, even detrimental to post-progression survival (96). The result highlights the need to identify predictive biomarkers that can discriminate the patients potentially benefited from bevacizumab. Although, circulating Tie2, ascites formation, BRCA1/2, homologous recombination deficiency and CD31 status can somewhat influence the efficacy, the identification of predictive biomarkers remains challenging and elusive (97, 98).

Moreover, cediranib, an inhibitor of VEGFR tyrosine kinase (VEGFR1-3), has exhibited limited efficacy and increased toxicity in various cancers, including ovarian cancer (99). Nonetheless, promising results have been observed in cediranib treatment for ovarian cancer patients, particularly regarding PFS when used in combination with chemotherapy and maintenance treatment (99). The combination of Poly ADP-ribose polymerase inhibitors and cediranib has demonstrated enhanced efficacy in inhibiting tumor proliferation and promoting the immune response within the tumor microenvironment, irrespective of HDR status, in a patient-derived xenograft model of ovarian cancer (100). The study conducted by Zhou et al. demonstrated that the plasma concentration of Tie2 serves as a reliable predictive biomarker for generic VEGF signaling inhibitor, including cediranib in ovarian cancer (101). Another murine *in vivo* investigation revealed that the TME intrinsically characterized with higher level of IL6 and JAK/STAT signaling counteracted the activity of cediranib (102). Besides, pazopanib (selective multi-targeted receptor tyrosine kinase inhibitor targeting VEGFR, FGFR, PDGFR, c-KIT, and c-Fms) effectively inhibits tumor growth and angiogenesis (103, 104). Although pazopanib is associated with adverse events such as neutropenia, it has demonstrated advantages in the treatment of refractory and platinum-resistant ovarian cancer, as well as in platinum-sensitive maintenance therapy (105–107). Additionally, nintedanib, another inhibitor targeting various tyrosine kinases, including PDGFR, FGFR, FLT3, and VEGFR, competitively suppresses non-receptor tyrosine kinases (108, 109). Nintedanib is known to cause common adverse events such as diarrhea (110). Nevertheless, phase 3 trials have exhibited remarkable therapeutic effects when nintedanib is combined with chemotherapy such as carboplatin and paclitaxel in ovarian cancer patients, although serious adverse gastrointestinal events accompany these positive effects (111–113).

In addition to VEGF signaling, there are alternative pathways targeted by angiogenesis inhibitors. ANGPT1/2 bind to the Tie-2 receptor and promote endothelial cell proliferation, survival, and motility. Trebananib, a non-VEGF-dependent anti-angiogenesis agent, binds to ANGPT1/2 and counteracts their action on the Tie-2 receptor, thereby effectively suppressing angiogenesis (114). Trebananib, when combined with paclitaxel, effectively improves the PFS of patients with recurrent ovarian cancer (115). In summary, targeting angiogenesis through VEGF/VEGFR signaling has shown promising results in ovarian cancer. Bevacizumab, cediranib, pazopanib, and nintedanib are among the agents that have been investigated in this context. However, challenges persist in terms of optimizing therapeutic responses, managing side effects, and identifying predictive biomarkers to facilitate better patient selection (116). Further research and clinical investigations are needed to improve the efficacies of anti-angiogenesis agents in ovarian cancer.

## 3 Anti-angiogenesis therapy reshapes the TME and enhances ICB efficacy

### 3.1 Angiogenesis and anti-tumor immunity

The antitumor immunity and ICB efficacy are regulated by the presence and status of tumor-infiltrating lymphocytes (TILs) as well as the process of angiogenesis (117–121). TILs are critical components of the immune response against tumors, and their presence is essential for successful tumor regression with ICB therapy, particularly with interventions targeting the PD-1/PD-L1 pathway. Angiogenesis plays a crucial role in the status of TILs and the response to ICI therapy. In the cancer-immunity cycle, the presentation of neoantigens initiates the formation of tumor-specific T cell clones, which then traffic to and infiltrate the tumor (122). TILs recognize these neoantigens and eliminate tumor cells in an immunosupportive TME (122). However, hyperactive angiogenesis leads to an immunosuppressive TME by affecting multiple processes of the immune response (123–125).

Abnormal angiogenesis negatively impacts the abundance and function of TILs (29). It creates physical barriers that impede T cell infiltration, such as leaky vessels with high interstitial fluid pressure and reduced expression of adhesion molecules like VCAM-1 (126). Additionally, the lack of adequate vasculature compromises the oxygen supply to the tumor, leading to hypoxia. Hypoxia, in turn, upregulates immunoinhibitory signals, including IL-6, IL-10, PD-L1, and IDO (19). Moreover, circulating VEGF hinders the maturation and activity of dendritic cells, facilitating immune evasion of tumor cells (127, 128). On the contrary, hyperactive angiogenesis promotes the expansion of immunohibitory lymphocytes. Tumor hypoxia resulting from abnormal blood vessels upregulates the expression of chemokines that recruit regulatory T cells (Tregs) into the tumor (129, 130). The hypoxic TME also induces the polarization of tumor-associated macrophages (TAMs) toward an M2-like phenotype, which further supports tumor growth and immune evasion (131). Additionally, Fas ligand expressed by tumor endothelial barriers selectively kills cytotoxic CD8^+^ T cells, while sparing Tregs due to increased cellular FLICE-inhibitory protein on Tregs (132). Angiogenesis exerts a multifaceted influence on tumor growth and immune evasion. Abnormal angiogenesis hampers TIL infiltration and function while promoting the recruitment and activity of pro-tumor lymphocytes.

### 3.2 The effects of anti-angiogenesis therapy on the TME

Anti-angiogenesis therapy aims to inhibit tumor angiogenesis and disrupt the abnormal tumor vasculature. Initially, these agents were developed to starve tumors by interfering with neo-vascularization (133). However, monotherapy with anti-angiogenic agents did not yield satisfactory results, as tumors developed mechanisms to tolerate hypoxia, leading to invasiveness and metastasis (134, 135). Nevertheless, anti-angiogenesis therapy has shown promise as a sensitizer when combined with other therapies. The concept of vessel normalization, describes the transient state of tumor vessels undergoing anti-angiogenesis treatment (133). In this state, tumor vessels undergo structural and functional changes, including enhanced perfusion, improved pericyte coverage, and relieved hypoxia. The duration and extent of vessel normalization depend on the treatment schedule and dose (136). Anti-angiogenesis therapy has also been shown to reprogram the TME from an immunoinhibitory to an immunosupportive state (137). Normalized tumor vasculature alleviates hypoxia, which can promote the polarization of TAM to an M1-like phenotype (136). Additionally, vessel normalization reduces the recruitment of Treg and myeloid-derived suppressor cells (MDSC) (138). Improved perfusion resulting from vessel normalization also downregulates hypoxia-mediated immunoinhibitory molecules, such as PD-L1 (139).

Preclinical studies have demonstrated the potential synergistic effect of combining ICB with anti-angiogenesis therapy (140–142). The crosstalk between the TME and angiogenesis suggests that anti-angiogenesis therapy may enhance the therapeutic effects of ICB. Various mechanisms have been proposed to explain this synergy. For example, anti-VEGF treatment can abrogate VEGF-induced immune checkpoint expression on intratumoral T cells (143). Furthermore, the induction of high endothelial venule (HEV) formation through combination therapy has been shown to promote T cell infiltration into tumors (39). Combining anti-angiogenesis therapy with ICI has shown promising results in preclinical studies, suggesting a potential strategy for improving the efficacy of immunotherapy. Further investigations are warranted to fully understand the mechanisms underlying the synergistic effects and to optimize the combination therapy approach.

## 4 Anti-angiogenesis combined with ICB therapy for ovarian cancer patients

In the phase 1 dose-escalation study NCT02298959, anti-angiogenesis agent ziv-aflibercept (recombinant fusion protein containing VEGF-binding domains developed based on VEGFR1/2) combined with anti-PD-1 antibody pembrolizumab exhibited potent antitumor activity in solid tumors (including ovarian cancer, renal cell carcinoma, colorectal cancer, and melanoma) with manageable safety profile (144). The median overall survival reached 12.5 months (90% CI 3.8 to 13.6) in the ovarian cancer subgroup (144). Besides, in the phase 1b trial NCT04236362, 34 platinum-resistant or refractory ovarian cancer patients were enrolled (145). In this clinical study, anti-angiogenesis agent anlotinib combined with anti-PD-1 antibody TQB2450 also showed encouraging antitumor activity (median PFS: 7.8 months; disease control rate: 97.1%; and objective response rate: 47.1%) (145).

Besides, in the phase 1b study NCT01633970, patients with platinum-resistant ovarian cancer benefited from atezolizumab and bevacizumab treatment (146). This combination therapy achieved a response rate of 15%, and all three patients with partial response had a long duration (11.3-18.9 months) (146). Furthermore, in the single-arm phase 2 study NCT02873962, nivolumab combined with bevacizumab showed promising antitumor potential for relapsed ovarian cancer (overall response rate: 28.9%; 95%CI 15.4%-45.9%), especially for platinum-sensitive patients (overall response rate: 40.0%, 95%CI 19.1%-64.0%) (147). Howbeit, combination treatment might predispose patients to cumulative adverse effects. Therefore, optimizing sequencing of administration of ICB and anti-angiogenesis agents is of importance. Intriguingly, due to the specific mechanism that anti-angiogenesis normalizes the tumor vasculature, a lower dosage of ICB can achieve the same efficacy as before (148). Generally, anti-angiogenesis combined with ICB therapy is a promising strategy for ovarian cancer, and more clinical studies are ongoing to further confirm the efficacy of this combination therapy.

## 5 Perspective

Angiogenesis, the formation of new blood vessels, plays a crucial role in tumor growth and metastasis. The VEGF/VEGFR and ANGPT/TIE systems are key players in the regulation of angiogenesis. Anti-angiogenesis therapy aims to disrupt tumor angiogenesis by targeting specific factors or receptors involved in this process. Bevacizumab, cediranib, pazopanib, and nintedanib are among the agents that have been investigated in the context of ovarian cancer treatment. While these agents have shown clinical efficacy, challenges remain in terms of optimizing therapeutic responses, managing side effects, and identifying predictive biomarkers. The combination of anti-angiogenesis therapy with ICB has shown promising synergistic effects in preclinical and clinical studies. This combination therapy has demonstrated the ability to increase the ratio of anti-tumor to pro-tumor immune cells, downregulate the expression of multiple immune checkpoints, and reshape the TME. The TME plays a critical role in response to ICB therapy, and angiogenesis has been found to have a significant impact on the status of TILs and the efficacy of ICB. Abnormal angiogenesis creates an immunosuppressive TME, hindering TIL infiltration and function while promoting the abundance of pro-tumor lymphocytes. Anti-angiogenesis therapy can normalize tumor vasculature, alleviate hypoxia, and reprogram the TME to an immunosupportive state.

Clinical trials investigating the combination of ICB and anti-angiogenesis therapy in ovarian cancer have shown encouraging outcomes. The combination has demonstrated improved PFS and overall survival compared to monotherapy. However, challenges remain in terms of patient selection, optimizing treatment regimens, and managing potential side effects. Further research is needed to identify predictive biomarkers that can guide treatment decisions and enhance therapeutic responses. Additionally, efforts should be made to develop more potent and specific anti-angiogenesis agents and explore novel targets in the angiogenesis pathway.

## 6 Conclusion

Ovarian cancer remains a significant health concern for women worldwide, and novel treatment strategies are urgently needed to improve patient outcomes. The combination of immune checkpoint blockade and anti-angiogenesis therapy has emerged as a promising approach for the treatment of ovarian cancer. Anti-angiogenesis therapy disrupts tumor angiogenesis and reshapes the tumor microenvironment, leading to improved infiltration and function of tumor-infiltrating lymphocytes. The combination therapy has demonstrated promising results in preclinical and clinical studies, with improved PFS and overall survival. Further research and clinical investigations are needed to address these challenges and unlock the full potential of the combination of ICB and anti-angiogenesis therapy in ovarian cancer. Efforts should be made to develop more potent and specific anti-angiogenesis agents and explore novel targets in the angiogenesis pathways. With continued advancements in understanding the complex interplay between angiogenesis and the immune response, this combination therapy holds promise in revolutionizing the treatment landscape for ovarian cancer.

## Author contributions

TL: Conceptualization, Supervision, Writing – review & editing, Project administration. PY: Investigation, Writing – original draft. YW: Investigation, Writing – review & editing. DY: Investigation, Writing – review & editing. YS: Investigation, Writing – review & editing. SQ: Conceptualization, Project administration, Supervision, Writing – review & editing.
